# Effect of atorvastatin on subclinical atherosclerosis in virally-suppressed HIV-infected patients with CMV seropositivity: a randomized double-blind placebo-controlled trial

**DOI:** 10.12688/f1000research.28262.2

**Published:** 2023-09-15

**Authors:** Evy Yunihastuti, Lusiani Rusdi, Muhammad Syahrir Azizi, Riwanti Estiasari, Chyntia Olivia Maurine Jasirwan, Endah Ayu T. Wulandari, Dyah Purnamasari, Mutiara Shinta Noviar, Sally Aman Nasution

**Affiliations:** 1 Allergy and Clinical Immunology Division, Internal Medicine Department, University of Indonesia Faculty of Medicine; Dr Cipto Mangunkusumo Hospital, Jakarta, 10430, Indonesia; 2 HIV Integrated Clinic, Dr Cipto Mangunkusumo Hospital, Jakarta, 10430, Indonesia; 3 Cardiology Division, Internal Medicine Department, University of Indonesia Faculty of Medicine; Dr Cipto Mangunkusumo Hospital, Jakarta, 10430, Indonesia; 4 Neurology Department, University of Indonesia Faculty of Medicine; Dr Cipto Mangunkusumo Hospital, Jakarta, 10430, Indonesia; 5 Hepatobiliary Division, Internal Medicine Department, University of Indonesia Faculty of Medicine; Dr Cipto Mangunkusumo Hospital, Jakarta, 10430, Indonesia; 6 Dentistry Department, University of Indonesia Faculty of Medicine, Jakarta, 10430, Indonesia; 7 Metabolic Endocrine Division, Internal Medicine Department, University of Indonesia Faculty of Medicine; Dr Cipto Mangunkusumo Hospital, Jakarta, 10430, Indonesia

**Keywords:** HIV, atherosclerosis, atorvastatin, cytomegalovirus, non alcoholic fatty liver, cognitive dysfunction, periodontitis

## Abstract

**Background: **Persistent immune activation and inflammation in HIV-infection are linked to excess cardiovascular risk and other non-communicable diseases. Periodic asymptomatic CMV-reactivity in HIV infected patients over a lifetime may contribute to non-AIDS defining morbidity. Despite undetectable levels of HIV and CMV, these patients continue to have increased levels of biomarkers and immune activations. Statin administration is thought to reduce subclinical atherosclerosis by decreasing LDL-C levels. It may also add beneficial effects against CMV infection.

**Methods: **We are conducting a double-blind placebo-controlled trial in which patients are randomized to receive either atorvastatin or placebo with a ratio of 1:1. This trial aims to study the effect of atorvastatin in statin-naive virally-suppressed HIV-infected patients with stable ART and CMV seropositivity on carotid intima media thickness (CIMT), tool that evaluates subclinical atherosclerosis. The study recruits 80 patients at HIV integrated care unit of Cipto Mangunkusumo hospital. All eligible subjects have CIMT evaluation as primary outcome, along with flow mediated vasodilatation (FMD), liver fibrosis and steatosis evaluation, fasting lipid, neurocognitive test, community periodontal index (CPI), and residual immune activation as secondary outcomes in 48 weeks.

**Ethics and dissemination: **This study has received an ethical approval from Health Research Ethics Commitee–Universitas Indonesia and Cipto Mangunkusumo Hospital. Before joining the study, all participants fill in an informed consent form. At the end of study analysis, the trial results will be published and disseminated in peer-reviewed journals.

**Discussion: **The main purpose of our study is to evaluate the effect of atorvastatin administration on CIMT changes in statin naïve virally suppressed HIV-infected patients with stable ART and CMV seropositivity

**Registration: **ClinicalTrials.gov ID NCT04101136; registered on 24 September 2019.

## Introduction

Highly active antiretroviral therapy (HAART) has contributed to significant reduction in AIDS related morbidity and mortality in HIV-infected patients ^1^. Increased longevity in HIV patients results on increased incidence of chronic non-communicable diseases (NCDs), particularly cardiovascular disease that has come out as an important cause of morbidity and mortality. Cardiovascular disease and other NCDs are associated with persistent inflammation and chronic immune activation that are still ongoing even in the state of viral suppression ^2^. Sustained low-level inflammation cause irritation to the blood vessel, promoting plaque growth, loosening the plaque, and triggering blood clots. This occurrence is the primary cause of strokes and heart attacks ^2^. 

There is accumulated evidence that some infectious agents, such as cytomegalovirus (CMV) and varicella zoster virus (VZV), may accelerate the course of atherosclerotic disease in HIV-infected patients ^3^. Cytomegalovirus seropositivity is commonly found in the Indonesian population. Prevalence of CMV seropositivity in pre-marital women is 78.9% while in blood donors is 98.23% ^4, 5^. Periodic asymptomatic CMV-reactivity in HIV-infected patients over a lifetime may play a part in non-AIDS defining morbidity, including cardiovascular disease, neurocognitive impairment, renal disease, and cancer ^6^.

Subclinical atherosclerosis has been described in several studies on HIV-infected patients but no special treatment has been confirmed yet. There are some traditional and non-traditional factors that may contribute to the acceleration of atherosclerotic process in HIV patient, such as dyslipidemia, ART-related dyslipidemia, and HIV-associated immune activation and inflammation.

Carotid intima media thickness (CIMT) is known to be the surrogate marker for subclinical atherosclerosis ^6, 7^. The direct correlation between CIMT and atherosclerosis among HIV-positive patients has been established in several studies ^8^. A prospective study conducted by Hsu R *et al.* described that greater CIMT was found in HIV-positive patients compared to that in HIV-negative patients, despite of viral suppression, lipid, and hypertension control ^9^. That study is parallel with our previous study which confirmed the slight rise of CIMT in ART-naïve patients 12 months after commencing ART. The CMV burden measured by using CMV IE level after ART admission was also correlated with right CIMT ^10^. We also found that CMV antibody level was inversely correlated with the Z score of cognitive result before ART admission ^11^.

Besides CIMT, FMD has also gained more attention in recent years despite its tight procedure. Flow-mediated dilatation describes any vasodilatation of an artery following an increase in luminal blood flow and internal-wall shear stress ^12^. The imbalance between vasoconstrictor and vasodilator factors lead to endothelial dysfunction. In addition, the release of inflammatory mediators and altered local shear forces may increase the synthesis of endothelial derived reactive oxygen species (ROS) ^13^. However, the correlation of FMD value in HIV patients remains a controversy ^14^. A small study conducted by Lebech AM *et al.* revealed no significant difference between FMD value among HIV-infected patients with a low cerebrovascular disease (CVD) risk compared to controls ^15^. Another study on population at risk of cardiovascular diseases stated that failure in FMD improvement after six months of antihypertensive therapy is an independent predictor of coronary events for the next five years ^16^. In a longitudinal study on ART-naïve patients, a considerable decrease in vasomotor function was found on FMD examination five weeks after ART initiation, which could be induced by immune reconstitution. This endothelial function decrease is supported by a handful of studies ^17– 19^.

Several studies have linked cardiovascular disease with oral inflammation, including periodontitis in the general population. However, its underlying mechanisms and cause-effect relationship remain unclear ^20, 21^. A report in 1989 demonstrated a link between periodontitis and myocardial infarction. Periodontitis has now been linked with greater CIMT and impaired FMD ^21^. Moreover, cardiovascular disease and periodontitis share predisposing factors, such as age, low socioeconomic and educational background, poor oral hygiene, smoking habits, and inflammatory genotypes ^22, 23^. HIV infection is thought to aggravate the existing periodontitis. This is supported by a recent study in the Netherlands on ART stable patients that linked the co-existence of HIV and periodontitis with higher risk of age-related diseases (cardiovascular diseases, autoimmune diseases, and diabetes mellitus) ^24^. In the general population, higher titers of the anti-CMV antibodies are proportionally correlated with the severity of the periodontal lesions and a subset of cases have detectable CMV DNA in affected tissues ^25^. Biofilm bacteria may initially induce gingivitis, which later results on consequent inflammation that may reactivate latent CMV (possibly from acinar cells) whilst the macrophage drawn into the periodontium may include cells infected with the virus, thus it is able to transfer it to somatic cells ^26^.

Non-alcoholic Fatty Liver Disease (NAFLD), another NCD that is common in HIV-infected patients, is also closely related to cardiovascular disease through different immune and metabolic processes. Patients with fatty liver disease are present with increased visceral adipose tissue (AT) secondary to inadequate intake of food. This causes a dysfunction in the AT generating insulin resistance, thereby increasing lipolysis and resulting in a greater release of free fatty acids (FFAs) into the bloodstream ^27^. Others suggest its relation to high concentration of apoprotein B (APOB) containing atherogenic lipoproteins. These are formed mainly by the process of increased hepatic production of non-high-density lipoprotein (non-HDL) cholesterol in order to decrease cholesterol toxicity from the diet ^28^. Inflammatory factors, pro-coagulation factors, greater formation of stress molecules, such as ROS and ceramides, also favor the progression of NAFLD and increase cardiovascular risk ^29^. The rising evidence of the association between NAFLD and subclinical CVD may suggest that NAFLD is not only a marker but may also be actively involved in pathogenesis of cardiovascular disease ^30^.

Statins, which were first extracted from *Pythim sp., Penicillium sp.*, and *Aspergillus sp*., are inhibitors of hydroxyl-3-methylglutaryl coenzyme A. Initially, the target of statin therapy was to reduce cholesterol levels. Recent studies showed that the use of statin was also associated with other atherogenic lipid particles reduction, such as oxidized LDL and phospholipase associated with lipoprotein ^31^. Statins remain the primary and secondary key of CVD prevention by improving endothelial function, slowing the progression of atherosclerosis and stabilizing atherosclerotic plaque. In the HIV population, decreased oxidized LDL is independently associated with a decrease in important markers of subclinical atherosclerosis, such as coronary plaque and CIMT. The dual mechanism of statin use reduces LDL cholesterol, and modifies inflammatory responses, antioxidant effects, antithrombic effects (clotting processes), and removal of blood vessel plaques, thereby preventing cholesterol build-up and muscle proliferation ^32^. Treating dyslipidemia with statins has been challenging in HIV-infected patients due to potential drug interaction between statins and antiretroviral drugs as both drugs are metabolized by cytochrome P450. A study comparing pitavastatin and pravastatin in patients using protease inhibitor shows higher virological failure in pravastatin group ^33^. However, pitavastatin is expensive as it is not available in generic formulation. Atorvastatin, another moderate-intensity statin, is available in generic formulation and is widely used in Indonesia. This drug is known to have no interaction with current antiretroviral drugs used in Indonesia. Seropositivity to CMV may jointly predict increased mortality rate in patients with coronary heart disease. Statin therapy is known to decrease lipid level and also has an anti-inflammatory effect. A study shows that statin reduces mortality rate among CMV-infected patients with coronary artery disease ^34^. An *in vitro* study demonstrates the broad anti-CMV activity of statins. All statin doses are found to dependently reduce CMV titers in human aortic endothelial cells. These findings provide new insight into beneficial effects of statins ^35^. The use of atorvastatin, a cheap and widely accessible drug in Indonesia, has not been specifically studied in term of improving cardiovascular outcome in HIV-infected patients under ART.

We conduct a randomized double-blind placebo-controlled trial that primarily aims to evaluate the effect of atorvastatin on carotid intima media thickness (CIMT) in virally-suppressed HIV-infected patients with CMV seropositivity. Secondly, this trial also evaluates the other marker values, such as FMD, liver fibrosis and steatosis, fasting lipid, neurocognitive test, CPI, and residual immune activation as secondary outcome for 48 weeks.

## Protocol

### Participants and interventions

This study uses randomized control trial in which subjects are assigned to a double blind randomized with placebo-controlled clinical trial study with atorvastatin 20 mg in virally-suppressed HIV-infected patients.

### Study setting and sample size

The study is taking place in a single center, the HIV integrated clinic Cipto Mangunkusumo Hospital, Jakarta. All patients receive government free antiretroviral program according to Indonesian guidelines. The first-line regimen consists of two nucleoside reverse transciptase inhibitors (NRTIs) and one non-nucleoside reverse transcriptase inhibitor (NNRTI), and the second-line regimen of two NRTIs and one protease inhibitor (PI). The majority of patients belong to urban and mainly low- to middle-income families living in Jakarta. Assuming that the effect size is 0.1, standard deviation is 0.12, and the use of 2 sample t-test, the sample size needed for 90% power and 5% significant level is 32 per arm. With an additional 20% drop out rate, each arm (placebo and control group) will need 40 participants.

### Eligibility criteria and recruitment

Patients, men and women aged between 20 to 45 years old, using stable ART (no adverse drug reactions, no pregnancy or current illnesses, understand lifelong adherence, and evidence of successful treatment) for at least a year with positive CMV IgG and viral load of HIV RNA <50 copies/mL are recruited to the study. Viral load, anti-CMV antibody, and lipid panel tests are done before defining the eligibility criteria. Participants are also required to not use any statin therapy 6 weeks before enrollment. Patients with the following conditions are excluded from the study:

- patients undergoing hepatitis C DAA therapy
- decompensated cirrhosis or acute liver failure
- history of coronary artery disease
- history of diabetes mellitus
- history of brain infection, epilepsy, or stroke
- history of rhabdomyolysis or myopathy
- pregnant or breastfeeding during study
- severe depression
- patients using statin therapy in the 6 weeks prior to the study
- history of statin hypersensitivity
- patients with a Framingham Risk Score above 10% within LDL ≥130

### Interventions and participant timeline

**Figure 1.**
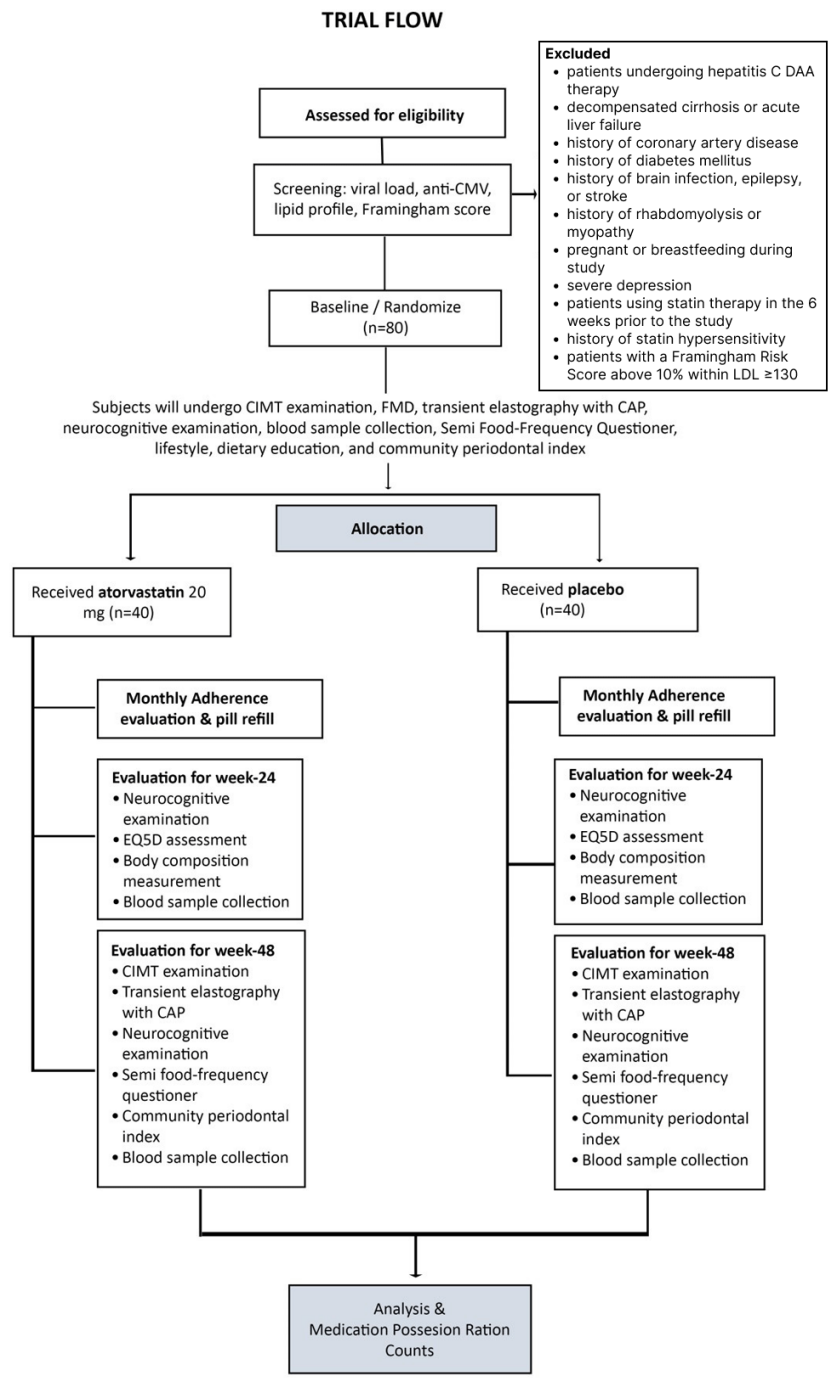
Study trial flow diagram. CONSORT diagram of the anticipated number of participants that need to be screened in order to reach a target of 80 participants randomized to atorvastatin or placebo.

All potential participants receive an explanation about the purpose and method of the study and are informed of their rights to stop or refuse to take part in the study. A special mobile number is available for potential participants to obtain additional information should they wish. All potential participants are contacted again to schedule an appointment if they agree to continue with all of the procedures. After screening is completed, they will be interviewed and examined at the clinic area, which includes CIMT, FMD, and neurocognitive evaluation. The oral examination is conducted at the dentistry clinic and the transient elastography examination will be conducted at the hepatology clinic. Figure 1 presents a CONSORT diagram of the anticipated number of participants needed to be screened in order to reach a target of 80 participants randomized to atorvastatin or placebo.

Participants receive either 48 weeks of 20 mg atorvastatin or placebo once daily. We use oral atorvastatin 20 mg capsule in generic form and a placebo capsule prepared by the hospital pharmacist, composed of starch which is similar to atorvastatin capsule in size, shape, and color. Packagings are identical in appearance for both atorvastatin and the placebo. CMV = cytomegalovirus; DAA = direct-acting antiviral; LDL = low density lipid; CIMT = carotid intima media thickness; FMD = flow mediated vasodilatation; CAP = controlled attenuation parameter.

### Study outcomes

The primary aim of this study is to compare the effect of 48 weeks of 20 mg daily atorvastatin versus placebo on CIMT in virally suppressed HIV patients with CMV seropositivity. Other aims are to assess:

- the interaction between baseline CMV copy number and the effect of atorvastatin on CIMT, FMD, neurocognitive function, liver fibrosis and steatosis, lipid profile, and immune activation biomarkers (sCD14, VCAM, ICAM, hsCRP, and β2M) changes in 48 weeks.
- the changes of lipid, liver fibrosis and steatosis, neurocognitive, several immune activation biomarkers, and CPI in virally suppressed HIV patients with CMV seropositivity given 48 weeks of 20 mg daily atorvastatin compared to placebo

### Assignment of interventions

Participants who meet the inclusion criteria are randomized into atorvastatin groups and placebo groups with a ratio of 1:1 (40 subjects per group). Randomization using STATA version 14.2 based on randomization of permutation blocks with block 4 sizes is carried out by statistical analysts. The administration of study drug is home-based participant self-administration. Participants are asked to swallow the whole capsule without chewing or breaking it. The participants get the medication supply every month along with the prescription of antiretroviral drugs.

### Masking

The code of each participant is given to pharmacists who are involved in the study. The pharmacists keep the code in the sealed envelope. Researchers, doctors, and participants do not know the results of randomization. The drug and placebo capsules are administered to patients by a staff member who is privy to the treatment. Each participant should return the unused capsules every month. At the end of the study, the pharmacist and related staff members will count the Medication Possession Ratio (MPR). The study will be discontinued if:

- the participant withdraws informed consent
- the participant develops significant toxicity, intolerance or allergy associated with study drug that causes a risk to participant’s life
- the participant is admitted to hospital for other morbidity that is incompatible with further atorvastatin treatment
- blinding is revealed. Unblinding will also be available if a clinician believes that clinical management depends importantly on knowledge of whether the patient is currently receiving statin or placebo.

### Data collection and management

Subjects who meet eligibility criteria are enrolled and have a baseline visit (week 0) within 6 weeks following the screening visit. Venous blood sample are collected for fasting blood glucose, ALT, CMV DNA, hsCRP, sCD14, VCAM, ICAM, and β2M examinations.

Each patient has a CIMT and FMD evaluation performed by a trained cardiologist using B mode ultrasonography. CIMT is defined as a double-line pattern visualized by echo 2D on both walls of the common carotid artery (CCA) in a longitudinal view. Two parallel lines (leading edges of two anatomical boundaries) form it: the lumen-intima and media-adventitia interfaces. High-resolution B-mode ultrasound images of the right and left common carotid artery will be used to measure carotid intima-media thickness. The patient is scanned in the supine position using 7–12 MHz linear array transducer (Philips Affiniti 70C, Phillips, UK). CIMT is calculated using both manual and semi-automated (Automated; QLAB, Phillips, UK) methods. Manual CIMT measurements are recorded from the far wall at 1 cm, 1.5 cm, and 2 cm intervals proximal to the carotid bulb. Automated measurements are also recorded from the far wall, using the same image, from the identical 1 cm section proximal to the carotid bulb. The carotid bulb is defined as the point where the far wall deviated from the parallel plane of the distal CCA. Mean manual and automated cIMT measurements for the right and left CCA are calculated from three consecutive cardiac cycles.

FMD is assessed in the brachial artery by ultrasonography. The participant has to abstain from exercise (≥12 hr), caffeine (≥12 hr), smoking or smoke exposure (≥12 hr), vitamin supplementation (>72 hr), and any medication (≥4 hr half-lives of the drug, non-steroidal anti-inflammatory agents for 1 day and aspirins for 3 days). We ensure that the participant is under fasting conditions or has only consumed low-fat meals. The measurements are performed on the right arm when the patient is in the supine position, after 10 to 20 minutes of rest in the supine position ^36, 37^ The brachial artery is scanned longitudinally just above the antecubital crease with a linear multifrequency 5- to 12-MHz transducer B-mode. The diameter of the brachial artery is measured on the interface between media and adventitia of the anterior and posterior wall as a baseline. Gain settings are optimized to identify the lumen and the vessel wall interfaces and could not be modified during the examination. Hyperemia is induced by inflation of a pneumatic cuff of 200-250 mm Hg for 5 minutes on the most proximal portion of the forearm. Arterial diameter measurement is repeated just before (30-60 seconds) deflation of the cuff, then just after (30 to 60 seconds) sudden deflation of the cuff. Tracings is recorded on image and file and are read by two investigators (MS, LR) who are unaware of the participant’s clinical data. The measurements of basal and posthyperemia diameter will be used for the analysis. Flow-mediated vasodilation is expressed as the relative increase in brachial artery diameter during hyperemia and defined as 100× [(posthyperemia diameter−basal diameter)/basal diameter].

Transient elastography with CAP will be done by trained hepatologist using Fibroscan (Echosens®) device. The criteria for successful examination are 10 shots and an interquartile range (IQR) for liver stiffness of less than 20% of the median value. The cut-off value for NAFLD diagnosis is CAP measurement of above 238 dB/m and/or fibrosis score higher than 7.1 kPa ^38^.

A trained neurologist performs the neurocognitive evaluation. The cognitive tests are performed in week 0, week 24, and week 48. This study uses tests that are sensitive to domain most affected in HIV but can be delivered in relatively short time. This study uses Trail Making Test (TMT) A and B to measure executive function, Symbol Digit Modalities Test (SDMT) for speed of information processing, and Brief Visuospatial Memory Test Revised for learning and memory. These tests have already been validated in Indonesia and can be administered by non-neuropsychologists who have been trained ^39^. To determine cognitive impairment, we use local normative data that match age and education level.

A trained dentist will assess periodontitis using CPI, which was modified by the World Health Organization (WHO). This assessment uses a 0.5 mm ball tip periodontal probe with black band markers at 3.5, 5.5, 8.5, and 11.5 mm, and involve 10 teeth (17, 16, 11, 26, 27, 37, 36, 31, 46, and 47). The score ranges from 0 for healthy periodontal to score 4 for periodontal pocket ≥6 mm ^40, 41^. Eligibility screen, informed consent, and physical examinations will be done within a week before taking study drugs. All other examinations will be done just before taking study drugs (week 0) and at the end of this study (week 48), except for neurocognitive and blood collection, which are done in baseline, in week 24, and at the end of this study in week 48, as seen in Table 1.

**Table 1.** Schedule of enrollment, intervention, and assessment.

	STUDY PERIOD														
	Screening and Enrollment	Randomization	Post-randomization												
TIMEPOINT (WEEK)	*-w _1_*	0	*w _4_*	*w _8_*	*w _12_*	*w _16_*	*w _20_*	*w _24_*	*w _28_*	*w _32_*	*w _36_*	*w _40_*	*w _44_*	*w _48_*	Extra ^*^
**SCREENING AND ENROLLMENT**															
Eligibility screen	X														
Informed consent	X														
Randomization		X													
**ASSESSMENT & EVALUATION**															
CIMT & FMD Examination		X												X	
Transient elastography and CAP		X												X	
Neurocognitive examination		X						X						X	
Community Periodontal Index		X												X	
Blood collection (fasting glucose, ALT, CMV DNA, hsCRP, sCD14, VCAM, ICAM, β2M examinations)		X						X						X	X
Study drug (atorvastatin or placebo)		X	X	X	X	X	X	X	X	X	X	X	X	X	
Physical Assesment	X	X						X						X	
Medication Possession Ratio														X	

W = week; *extra visit will be done when adverse event occurs during the study.

At the end study, a total drug consumption adherence will be assessed using MPR. Meanwhile, each patient has monthly clinical follow-ups as standard of care in the clinic. Study follow-up visits include clinical and laboratory procedures as presented in Table 1. MPR is the sum of the days’ supply for all fills of a given drug in a particular time period, divided by the number of days in the time period ^42^.

Data management are managed in the HIV Integrated Care Unit, Cipto Mangunkusumo Hospital. All research staff have overall responsibility for maintaining the study CRFs. Corrections to the paper CRF documents must be made by striking through the incorrect entry with a single line (taking care not to obliterate or render the original entry illegible) and entering the correct information adjacent to the incorrect entry. Corrections to paper CRFs must be initialed and dated by the person making the correction. The researchers are responsible for maintaining accurate, complete, and up-to-date records. These forms are to be completed on an on-going basis during the course of the study.

### Statistical analysis

Statistical analyses will be performed with SPSS (version 21). Subjects will be analyzed in the group to which they are randomized, regardless of the treatment received. A T test will be used to compare the mean CIMT changes of virally supressed HIV patients with CMV seropositivity in the atorvastatin group versus the placebo group between the baseline and week 48 to analyze primary endpoints. Meanwhile, secondary endpoints will be analyzed using linear regression with interaction to assess the magnitude of change in the effect of atorvastatin on CIMT associated with the baseline CMV copy number, and a proportional odds logistic regression model will be used to estimate the odds ratios of CPI in treating an increased level of CIMT. Other continuous endpoints will be analyzed using a 2 sample T-test. Missing data will be resolved by comparing subject characteristics in the drop out group with the remaining participants. If there is no difference in characteristics it would assumed that the missing data is at random.

### Monitoring

The study is to be monitored by Cipto Mangunkusumo Hospital Research Unit. Research records for all study subjects including CRFs, laboratory, and other investigation data/results are to be maintained by the investigator in secure storage for five years. These records are to be maintained in compliance with all designated IRBs requirements. It is the investigator’s responsibility to retain copies of these study records until notified in that they can be destroyed. Any severe adverse event or unintended effects of trial intervention will be reported and assessed by the ethics committee.

### Patient and public involvement

Participants begin involvement in the trial when the eligibility criteria are fulfilled and screening examinations are surpassed. Clear information about the trial, including participant’s role, is explained by study team and is written on informed consent form. Each participant has his/her right to decline or withdraw from the trial. The participant also has the right to receive any information regarding test result done during the trial. One of the participants and experienced team member are given the role of patient recruitment.

### Ethics

This trial has been approved by Health Research Ethics Commitee–Universitas Indonesia and Cipto Mangunkusumo Hospital (HREC-FMUI/CMH) and registered in ClinicalTrials.gov (identification number 19-03-0272). Any amendments or modification regarding the protocol during the trial will be consulted and notified to HREC-FMUI/CMH. All participants are required to give their consent before joining the study. The consent form is written in Indonesian and is composed of the following details: participant’s name, date of birth, investigator contact, and explanation of the study purpose that is easily understood by non-medical personnel, course and duration of the study (as well as the freedom to withdraw at any time and guarantee of confidentiality), details of invasive procedures involved (blood collection), an explanation of blood remnant storage, the type of intervention (atorvastatin vs placebo), and possible side effects. The details of the informed consent form are explained by on-site research staff. Blood collections are collected by professional health workers and processed by lab analysts. The study team receives training on Good Clinical Practices (GCP) to ensure that sensitive research activities will be handled appropriately. The study maintains records of adverse events, as well as copies of the consent forms. All records are placed in a locked filing cabinet at the clinic that is only accessible by the research team. All team members are responsible for data security and record keeping. The datasets that will be used for analysis do not contain any identifying information, specifically, no names of participants are included in the datasets. Identifiers for the participants will be disposed of no more than three years after study completion. To protect confidentiality, identifiers are only accessible by the PI or project coordinator and are kept separated from other records with participants’ responses.

Participants presenting complaints or adverse event related to the trial will have an extra visit to medical personnel.

### Dissemination plan

At the end of study analysis, the trial results will submitted and published in peer-reviewed journals. Links of publication will be provided in the applicable trial register. Authorship for all publications will be based on the criteria defined by the International Committee of Medical Journal Editors.

## Discussion

This study is a double-blind and placebo-controlled trial. The main purpose of this study is to evaluate the effect of atorvastatin administration on CIMT changes in statin naïve virally suppressed HIV-infected patients without any comorbidities with stable ART and CMV seropositivity. To our knowledge this is the first study which is conducted in a population of this kind.

A study of statin-intervention with a 48 week follow-up comes with challenges and considerable limitations. First, the possibility of missed visits by study patients. Second, managing the follow-up of treatment and evaluation because this study involves multi-disciplinary specialists. Our solutions to avoid these problems is assigning a research assistant to regularly and preemptively remind patients and specialist teams to attend or carry out scheduled procedures by phone or message. If the patients cannot be contacted, the research assistant will check the schedule of antiretroviral collection at the administration desk. Another challenge arises from the Covid-19 pandemic. We have faced the difficulty of conducting direct monitoring due to the implementation of social restrictions. Many HIV-infected patients using delivery services to get their antiretroviral drugs in order to maintain their medications. We also conduct online monitoring and maintain study drug medications using delivery services. When it comes to procedural examination, physical barriers are provided along with personal protective equipment.

In sum, if the results as agree with our hypothesis, statin administration may be useful to prevent negative cardiovascular outcomes, especially the carotid intima medial thickness among virally suppressed HIV-infected patients and CMV seropositivity.

## Data Availability

No data are associated with this article Figshare: SPIRIT checklist for “Effect of atorvastatin on subclinical atherosclerosis in virally-suppressed HIV-infected patients with CMV seropositivity: A randomized double-blind placebo-controlled trial”. https://doi.org/10.6084/m9.figshare.13604831.v1 ^43^. Data are available under the terms of the Creative Commons Attribution 4.0 International license (CC-BY 4.0).
