# Supplementary material for: Effect of atorvastatin on subclinical atherosclerosis in virally-suppressed HIV-infected patients with CMV seropositivity: a randomized double-blind placebo-controlled trial
Source: F1000Res. 2023 Sep 15;10:151. Originally published 2021 Feb 26. [Version 2] doi: 10.12688/f1000research.28262.2 (PMC10523096; doi:10.12688/f1000research.28262.2)
Supplement: Peer review history

# Reviewer report: Reviewer response for version 2

**DOI:** 10.5256/f1000research.155979.r207026

**Published:** 2023-09-26

**Authors:** Vicente Estrada

**Affiliations:** 1 San Carlos Clinical Hospital, IDISSC, Complutense University, Madrid, Spain

I agree that the number of patients included in the study may seem small, but it is sufficient considering it is a clinical trial with the highest quality of scientific evidence. The reasons for excluding patients over the age of 45 are not completely conclusive, but based on the available information, I believe the study can still be accepted for indexing. Moreover, this study provides valuable information on a topic with limited published research

Is the study design appropriate for the research question?

Partly

Is the rationale for, and objectives of, the study clearly described?

Yes

Are sufficient details of the methods provided to allow replication by others?

Yes

Are the datasets clearly presented in a useable and accessible format?

Partly

Reviewer Expertise:

NA

I confirm that I have read this submission and believe that I have an appropriate level of expertise to confirm that it is of an acceptable scientific standard.

# Reviewer report: Reviewer response for version 2

**DOI:** 10.5256/f1000research.155979.r207025

**Published:** 2023-09-22

**Authors:** Rudi Wisaksana

**Affiliations:** 1 Head of HIV Research Group, Research Center for Care and Control of Infectious Disease, Universitas Padjadjaran, Bandung, Indonesia

I have no further comments

Is the study design appropriate for the research question?

Partly

Is the rationale for, and objectives of, the study clearly described?

Yes

Are sufficient details of the methods provided to allow replication by others?

Yes

Are the datasets clearly presented in a useable and accessible format?

Yes

Reviewer Expertise:

HIV

I confirm that I have read this submission and believe that I have an appropriate level of expertise to confirm that it is of an acceptable scientific standard.

# Reviewer report: Reviewer response for version 1

**DOI:** 10.5256/f1000research.31258.r129619

**Published:** 2023-06-06

**Authors:** Rudi Wisaksana

**Affiliations:** 1 Head of HIV Research Group, Research Center for Care and Control of Infectious Disease, Universitas Padjadjaran, Bandung, Indonesia

This article by Yunihastuti et. al. is an interesting attempt to answer intriguing questions regarding CVD prevention in people co-infected with HIV and CMV with low-risk CVD, in a major hospital in Indonesia.

 The study protocol was extensive with numerous markers evaluations. But the relatively short duration of observations and the limited number of study subjects might have an effect on the results.  

**Major:**

1. My major concern is that judgment on sample size and duration of the follow-up needed to show the effect of Atorvastatin on subclinical atherosclerosis was based on assumption only. The investigators should have considered this more carefully, given the large number of variables involved.
2. Other questions is regarding the study population. HIV-infected people still have ongoing low-level HIV viremia and low-level inflammation that exist even in virally-supressed persons. Why the investigators choose this population should be stated in the article.

**Minor:**

1. In the **Protocol** sections:
  1. *Participants, interventions, and outcomes.*
Comment: It was suggested that the subheading be changed to be *Participants and interventions *as there was another sub consisting of the study outcomes on the later page (page 6 of 11)
  2. *Study setting and sample-size.*
Comment: Is there any reference used regarding the SD value utilized in the sample size measurement? Or is it purely based on the investigator clinical assumption?
  3. *Eligibility and enrollment.*
Comments:
    - Can you provide the clear definition of ‘stable ART’ is?
    - Please add the limit period of the VL result date in the screening process will it be qualified as sourced documents to enroll the subject.
    - Do you hold any extra screening exam regarding the exclusion conditions in the case of subject having no related sourced documents prior to the screening process?
    - Please provide further details about how you define the existence of each exclusion conditions within the potential subjects and what kind of sourced documents will be relied on (maybe you can add bracket form at each exclusion conditions bullets e.g., history of diabetes {fasting glucose >125mg/dL}).
  4. *Interventions and participant timeline.*
Comment: I believe the sentence “Viral load, anti-CMV antibody, …… the eligibility criteria.” (Line 5-6) and the sentence “Participants are also ….. weeks before enrollment.” (Line 7-8) belongs to the prior sub and should be moved to be written in the *Eligibility and enrollment *sub.
  5. *Study outcomes.*
Comments:
    - Why did you choose those parameters used for the residual immune activation measurement among other parameters?
    - Please rephrase and eliminate the redundant words in the paragraph so it feels more clear and not repetitious.
  6. *Data collection and management.*
Comments:
    - Please use the same terminology in defining the study timeline. Which of the following is considered a baseline? The W-1 or W0?
    - Please use the same terminology in defining the study visit. Consistent with the unit whether it is using months or weeks.
2.   
**Figure 1**:
  1. In the box titled "Excluded”, there were consisted of 2 bullets only (“not meeting inclusion criteria and decline to participate”). On the other hand, in the prior sub it was mentioned that there were certain exclusion conditions and I believe it should be reflected in the diagram. May you add another bullet in the box considering this.
  2. Please add any footnotes regarding any defined shortened terms used in the diagram.
3. **Table 1**:
  1. Please add details about what laboratory examinations are done during the blood sample collection in each related study visit.
  2. I think it was more suitable to change the “Allocation” term into “Randomization”, thus the “Post-allocation” into “Post-randomization” and the “Enrollment” term into “Screening and Enrollment”.

Is the study design appropriate for the research question?

Partly

Is the rationale for, and objectives of, the study clearly described?

Yes

Are sufficient details of the methods provided to allow replication by others?

Yes

Are the datasets clearly presented in a useable and accessible format?

Yes

Reviewer Expertise:

HIV

I confirm that I have read this submission and believe that I have an appropriate level of expertise to confirm that it is of an acceptable scientific standard, however I have significant reservations, as outlined above.

## References

[1] : Effects of statin therapy on coronary artery plaque volume and high-risk plaque morphology in HIV-infected patients with subclinical atherosclerosis: a randomised, double-blind, placebo-controlled trial. *The Lancet HIV* .2015;2(2) : 10.1016/S2352-3018(14)00032-0 e52-e63 10.1016/S2352-3018(14)00032-0 PMC482082826424461

# Response

**Published:** 2023-09-13

**Authors:** Evy Yunihastuti

**Affiliations:** Dr. Cipto Mangunkusumo Hospital, Indonesia

Thank you for your comments,

 Major:

1. The sample size for randomized controlled trial was calculated according to the primary aim: to evaluate the effect of atorvastatin on carotid intima-media thickness in virally-suppressed HIV-infected patients with CMV seropositivity. The assumptions done were meant for the effect size (0.1) with a standard deviation of 0.12. We decided to use 90% for power and 5% for significant level for the sample size, resulting in 32 participants per arm. Considering an additional 20% drop in each arm, a total of 40 participants in each arm was needed.
2. We considered HIV-infected participants the target population due to their sustained inflammation. We included additional information regarding this population in the second paragraph of the introduction.

Minor:

1. Protocol sections:
  1. Participants, interventions, and outcomes
    1. Thank you for the suggestion. We have changed it into “Participants and Interventions”.
  2. Study setting and sample size
    1. Yes, we used a formula in order to determine the SD value in the sample size measurement. The formula is as follow: *n1=n2*=[(Z∝+Zβ)S/(x1-x2) *]* *2*  with n1 and n2 being sample size for group 1 and 2, Zα = 1.96, Zβ = 1.64, S for standard deviation, and x1-x2 for the minimum difference between the means that is considered meaningful.
  3. Eligibility and enrollment
    1. Stable ART is defined by “those who were receiving ART for at least 1 year with no adverse drug reactions requiring regular monitoring, no current illnesses or pregnancy, a good understanding of lifelong adherence, and evidence of treatment success”. ^1^
    2. The limit period for VL result date is 1 year for the screening process.
    3. The cholesterol level was checked for every participant before statin therapy was given. Other examinations were done not as part of the study protocol, but were taken from medical record or history taking.
    4. Each exclusion condition was evaluated from the medical record. Diabetes mellitus, severe depression, and other conditions were defined by the medical record or history taking.
  4. Interventions and participant timeline
    1. Thank you for the comment. We have moved it to the “Eligibility and enrollment” sub.
  5. Study outcomes
    1. We chose sCD14, VCAM, ICAM, hsCRP, and β2M as the residual immune activation parameters for these reasons below:
      1. sCD14 is expressed on macrophages as receptor for lipopolysaccharides on bacterial surfaces and has been reported to be higher in patients with residual HIV viral load than undetectable. Statin administration has the potency to reduce sCD14. ^2,3^
      2. VCAM is an endothelial activation found higher in viral-suppressed patients than in control. Increased expression of VCAM is associated with inflammation-related vascular complications. ^4^
      3. ICAM is expressed on endothelial cells and leukocytes at low levels but is stimulable to greater density by pro-inflammatory cytokines. ^5^ We hypothesize that statin may reduce ICAM by interfering with the ligand binding. ^6^
      4. hsCRP is a pentametric protein produced by the liver in response to IL-6 secretion and is a biomarker for early inflammation. Statin is thought to reduce CRP by regulating CRP formation directly in hepatocytes. ^7^
      5. β2M is a lymphocyte activation marker that increases in patients with tumors, renal dysfunction, immunoreactive conditions, or infections, including HIV. ^8^ Patients with more progressive disease have higher β2M levels, with highest in AIDS. ^9^
    2. We have modified the sentences so they are not redundant. Thank you for the input.
  6. Data collection and management
    1. The baseline of the study is W0.
    2. Thank you for the input. We have changed the unit with weeks.
2. Figure 1
  1. Thank you for the suggestion. We have added more bullets to the box.
  2. We have added footnotes to the diagram.
3. Table 1
  1. We have added the details.
  2. We have changed the terms accordingly, as suggested.

Reference:

1. Waldrop G, Doherty M, Vitoria M, Ford N. Stable patients and patients with advanced disease: consensus definitions to support sustained scale up of antiretroviral therapy. Trop Med Int Health. 2016 Sep;21(9):1124-30. doi: 10.1111/tmi.12746. Epub 2016 Jul 22. PMID: 27371814.
2. Falasca F, Di Carlo D, De Vito C, Bon I, d’Ettorre G, Fantauzzi A, et al. Evaluation of HIV-DNA and inflammatory markers in HIV-infected individuals with different viral load patterns. BMC Infect Dis. 2017;17: 581.
3. Chastain DB, Kayla RS, Daniel MR. Evidence-based review of statin use in patients with HIV on antiretroviral therapy. J Clin Transl Endocrinol. 2017; 8: 6-14.
4. Eckard AR, Yin J, Sara MD, et al. Effect of 24 weeks of statin therapy on systemic and vascular inflammation in HIV-infected subjects receiving antiretroviral therapy. J Infect Dis. 2014; 209(8): 1156-1164.
5. El-Gohary AM, Fawaz NA, Hassoba HM, Serwah A, Ali M. Soluble ICAM-1 in patients with chronic hepatitis C infection: a prognostic marker of disease activity. Egypt J Immunol. 2004;11: 109–119.
6. Videm V, M Albrigsten. Soluble ICAM-1 and VCAM-1 as markers og endothelial activation. Scandinavian Journal of Immunology. 2008; 67(5).
7. Montecucco F, Burger F, Pelli G, et al. Statins inhibit c-reactive protein-induced chemokine secretion, ICAM-1 upregulation and chemotaxis in adherent human monocytes. Rheumatology (Oxford). 2009; 48(3): 233-42.
8. Grady BPX, Nanlohy NM, van Baarle D. HCV monoinfection and HIV/HCV coinfection enhance T-cell immune senescence in injecting drug users early during infection. Immunity & Ageing. 2016 Dec 31;13(1):10.
9. Rao M, Sayal SK, Uppal SS, Gupta RM, Ohri VC, Banerjee S. BETA-2-MICROGLOBULIN LEVELS IN HUMAN-IMMUNODEFICIENCY VIRUS INFECTED SUBJECTS. Med J Armed Forces India. 1997 Oct;53(4):251-254. doi: 10.1016/S0377-1237(17)30746-3. Epub 2017 Jun 26. PMID: 28769505; PMCID: PMC5531080.

Thank you and best regards,

 Evy

# Reviewer report: Reviewer response for version 1

**DOI:** 10.5256/f1000research.31258.r97333

**Published:** 2021-12-01

**Authors:** Vicente Estrada

**Affiliations:** 1 San Carlos Clinical Hospital, IDISSC, Complutense University, Madrid, Spain

This manuscript is a research project that aims to analyze the effect of atorvastatin vs. placebo on carotid IMT variation over 48 weeks of treatment. The hypothesis is interesting, and the methods (double-blind, placebo-controlled) are correct.

 There are previous publications on this field that addresses the research hypothesis (PMID: 27203715 ^1^, PMID: 32353062 ^2^, in the general population PMID: 17384434 ^3^). Furthermore, the number of patients included seems low; estimates consider that atorvastatin will reduce IMT by 10% at 48 weeks, which seems a bit of an exaggerated estimate. Finally, the 48-week follow-up is scarce because the IMT variations need more time. The age >45 y exclusion criteria are not clearly explained.

 On the other hand, the hypothesis that atorvastatin will reduce the degree of steatosis after one year does not have a solid background. It has not shown definitive efficacy in the general population without being associated with vitamin E (PMID: 20842109 ^4^). On the other hand, I believe that it disperses the study's primary objective, and indeed it will not have statistical power to confirm the hypothesis.

Is the study design appropriate for the research question?

Partly

Is the rationale for, and objectives of, the study clearly described?

Yes

Are sufficient details of the methods provided to allow replication by others?

Yes

Are the datasets clearly presented in a useable and accessible format?

Partly

Reviewer Expertise:

HIV

I confirm that I have read this submission and believe that I have an appropriate level of expertise to confirm that it is of an acceptable scientific standard, however I have significant reservations, as outlined above.

## References

[1] : Rosuvastatin slows progression of subclinical atherosclerosis in patients with treated HIV infection. *AIDS* .2016;30(14) : 10.1097/QAD.0000000000001167 2195-2203 10.1097/QAD.0000000000001167 PMC500714227203715

[2] : Association between statin use, atherosclerosis, and mortality in HIV-infected adults. *PLoS One* .2020;15(4) : 10.1371/journal.pone.0232636 e0232636 10.1371/journal.pone.0232636 32353062PMC7192415

[3] : Effect of rosuvastatin on progression of carotid intima-media thickness in low-risk individuals with subclinical atherosclerosis: the METEOR Trial. *JAMA* .2007;297(12) : 10.1001/jama.297.12.1344 1344-53 10.1001/jama.297.12.1344 17384434

[4] : Atorvastatin and antioxidants for the treatment of nonalcoholic fatty liver disease: the St Francis Heart Study randomized clinical trial. *Am J Gastroenterol* .2011;106(1) : 10.1038/ajg.2010.299 71-7 10.1038/ajg.2010.299 20842109

# Response

**Published:** 2022-01-28

**Authors:** Evy Yunihastuti

**Affiliations:** Dr. Cipto Mangunkusumo Hospital, Indonesia

Thank you for your comments,

1. In our sample size calculation, we assume that the effect size is 0.1 with a standard deviation of 0.12. With the plan to use of 2 sample t-test, the sample size needed for 90% power and 5% significant level is 32 per arm. Assuming an additional 20% drop in each arm (placebo and control group), we need a minimum of 40 participants.
2. We exclude subjects >45 years because the highest PLHIV prevalence in Indonesia is 20-45 years and we want to see the effect of atorvastatin on subclinical atherosclerosis in virally-suppressed HIV-infected patients with CMV seropositivity. A previous study reported that CVD morbidity and mortality in PLHIV is higher above 45 years old than in negative controls. ^1^

Reference:

 Chastain DB, Stover KR, Riche DM. Evidence-based review of statin use in patients with HIV on antiretroviral therapy. J Clin Transl Endocrinol. 2017 Feb 22;8:6-14.

 Thank you and best regards,

 Evy
